# A Longitudinal Twin Study of the Direction of Effects between ADHD Symptoms and IQ

**DOI:** 10.1371/journal.pone.0124357

**Published:** 2015-04-15

**Authors:** Anna Sophie Rommel, Frühling Rijsdijk, Corina U. Greven, Philip Asherson, Jonna Kuntsi

**Affiliations:** 1 King’s College London, Medical Research Council Social, Genetic and Developmental Psychiatry Centre, Institute of Psychiatry, Psychology and Neuroscience, London, United Kingdom; 2 Radboud University Medical Centre, Department of Cognitive Neuroscience, Donders Institute for Brain, Cognition and Behaviour, Nijmegen, The Netherlands; 3 Karakter Child and Adolescent Psychiatry University Center, Nijmegen, The Netherlands; University of Hong Kong, HONG KONG

## Abstract

While the negative association between ADHD symptoms and IQ is well documented, our knowledge about the direction and aetiology of this association is limited. Here, we examine the association of ADHD symptoms with verbal and performance IQ longitudinally in a population-based sample of twins. In a population-based sample of 4,771 twin pairs, DSM-IV ADHD symptoms were obtained from the Conners’ Parent Rating Scale-Revised. Verbal (vocabulary) and performance (Raven’s Progressive Matrices) IQ were assessed online. ADHD symptom ratings and IQ scores were obtained at ages 12, 14 and 16 years. Making use of the genetic sensitivity and time-ordered nature of our data, we use a cross-lagged model to examine the direction of effects, while modelling the aetiologies of the association between ADHD symptoms with vocabulary and Raven’s scores over time. Although time-specific aetiological influences emerged for each trait at ages 14 and 16 years, the aetiological factors involved in the association between ADHD symptoms and IQ were stable over time. ADHD symptoms and IQ scores significantly predicted each other over time. ADHD symptoms at age 12 years were a significantly stronger predictor of vocabulary and Raven’s scores at age 14 years than vice versa, whereas no differential predictive effects emerged from age 14 to 16 years. The results suggest that ADHD symptoms may put adolescents at risk for decreased IQ scores. Persistent genetic influences seem to underlie the association of ADHD symptoms and IQ over time. Early intervention is likely to be key to reducing ADHD symptoms and the associated risk for lower IQ.

## Introduction

Attention-deficit/hyperactivity disorder (ADHD) is associated with lower mean IQ scores. The correlation between IQ and ADHD symptoms reported in general population samples ranges from—0.2 to—0.4 [1,2]. A meta-analysis of 123 studies estimated a 7–11-point difference in full-scale IQ between control individuals and individuals diagnosed with ADHD [3]. Although not significantly different, the effect size (ES) of verbal IQ (VIQ; ES = 0.67; 95% CI 0.58–0.76) was larger than that of performance IQ (PIQ; ES = 0.58; 95% CI 0.48–0.68). A large family-based study of 238 ADHD families (545 children) and 147 control families (271 children) also found that children diagnosed with ADHD and their affected and unaffected siblings had lower mean scores on VIQ, but not PIQ [4]. Moreover, lower VIQ than PIQ has been reported for adolescents diagnosed with ADHD [5].

In a clinical sample, children with IQs < 70 showed more severe ADHD symptoms than children with IQ > 70 [6]. Lower IQ is also associated with higher levels of externalising and behavioural problems in individuals with ADHD [7]. IQ has been shown to positively impact the response to pharmaceutical treatment in ADHD [8–11]. Therefore, IQ impacts on overall outcome in individuals with ADHD and is a good predictor of life success in the general population [12,13].

The heritability of ADHD symptoms is high at ~70% [14]. The extent of genetic factors influencing ADHD symptoms are relatively stable, although new as well as stable genetic influences are seen at different developmental stages [15–17]. For IQ, a developmental pattern is observed, whereby the extent of genetic influences on IQ gradually increases with age: heritability is estimated at around 30–60% in middle childhood and increases to 50–80% in adolescence and adulthood [18–20]. The largely genetic origin of the association of IQ and ADHD symptoms was initially shown in a large population-based sample of 5-year old twins, where 86% of the phenotypic correlation between ADHD symptom and IQ scores and 100% of the association between ADHD research diagnosis and IQ scores could be accounted for by genetic factors [1]. The genetic correlations, which indicate the degree to which genetic influences contributing to one trait are shared with genetic influences contributing to another trait, were r_A_ = -0.45 between IQ and ADHD symptoms and r_A_ = -0.59 between IQ and ADHD research diagnosis [1]. Significant genetic correlations have since been found in population-based twin samples of similar age [21,22], and in twin samples aged 7 to 10 years [2,23].

Although the shared genetic origins of and negative correlation between ADHD and IQ are well documented, little is known about the direction of effects or the genetic overlap between these variables at different developmental stages. The first objective of this study was to establish the developmental pattern of the association of ADHD symptoms with VIQ and PIQ in a population-based twin sample. While ADHD symptoms diminish in severity over the course of puberty (~ages 12–16 years [24]), individuals with high levels of ADHD symptoms are at increased risk of continuing problems related to ADHD during adolescence [25,26]. We are therefore, interested in the developmental pattern of the association of ADHD symptoms with VIQ and PIQ at ages 12, 14 and 16 years; a period of rapid developmental change for which both ADHD and IQ data were available in our large twin sample.

Previous research suggests a greater negative association between ADHD symptoms and VIQ than between ADHD symptoms and PIQ [3–5]. VIQ and PIQ are, thus, examined separately, allowing us to explore potential strengths and weaknesses associated with ADHD symptoms. The second aim was to examine the genetic and environmental aetiologies of the association of ADHD symptoms with VIQ and PIQ within time point, and their stability across time. Making use of the inherently time-ordered nature and genetic sensitivity of the data, we use a cross-lagged model to address these two objectives: namely, we establish the direction of effects, while modelling the genetic and environmental variance and covariance structure of the data separately for VIQ and PIQ.

## Methods

### Sample

Data came from the Twins Early Development Study (TEDS), a UK population-representative sample of twins born in England and Wales between 1994 and 1996 [27]. Zygosity of the twins was initially based on physical similarity ratings and later verified using genotyping. Participants were excluded following pre- or perinatal complications or if one or both twins suffered from any severe medical condition (e.g. autism spectrum disorder, cerebral palsy, Downs’ syndrome). Uncertain sex or zygosity and failure to provide information at recruitment were also exclusion criteria. After exclusion, 1,745 monozygotic (MZ) and 3,026 dizygotic (DZ) twin pairs were included in the model-fitting analysis. 46.1% of the participants were male. All twins (born in 1994, 1995 or 1996) were contacted simultaneously in order to simplify administration. Thus, twin ages ranged from 10.1 to 13.7 years of age at time point 1 (t_1_; mean age = 11.6 years, SD = 0.68), from 12.8 to 15.8 years of age at time point 2 (t_2_; mean age = 14.0 years, SD = 0.60) and from 15.8 to 17.3 years of age at time point 3 (t_3_; mean age = 16.5 years, SD = 0.27).

### Ethics Statement

Ethical approval was obtained from the King’s College London, Institute of Psychiatry, Psychology and Neuroscience ethics committee and all participants and/or their parents gave written consent for participation in this study.

### Measures

#### ADHD symptoms

ADHD symptoms were measured using the DSM-IV items on the Conners’ Parent Rating Scale-Revised [28]. The scores on the nine-item hyperactive-impulsive symptoms subscale were added to the scores on the nine-item inattentive symptoms subscale to form a total DSM-IV ADHD symptoms subscale. At t_1_, parent booklets enclosing the Conners’ Parent Rating Scale-Revised were sent in the post. At t_2_ and t_3_, similar parent booklets were sent to the families as online versions designed to mimic the paper version’s appearance.

#### General cognitive ability

All IQ assessments were administered online. See Haworth et al. [29] for details on how these web-based tests were created. A web-based multiple-choice version of the WISC-III vocabulary subtest was used to measure VIQ at t_1_ and t_2_. At t_3_, an online version of the Mill Hill Vocabulary scale was used as an indicator of VIQ because the WISC is not designed to measure intelligence beyond age 16 years. In both the WISC-III vocabulary subtest and the Mill Hill Vocabulary scale, the participant's task was to select the correct synonym for a word from a list of alternatives provided. Here, we refer to the outcome of these measures as vocabulary scores. PIQ was measured using a web-based version of the Raven’s Progressive Matrices test [30] at all three time points. At age 16 years, these web-based IQ tests were validated by comparing the online results for a subsample with their results on traditional paper-and-pencil tests. An average correlation of 0.72 between online and offline tests, administered 2 months apart, was found, providing support for the validity of these web-based tests [27].

Widespread access to internet connections in the UK has made online testing an attractive possibility for collecting data on large samples. Studies have shown that web-based findings are consistent with findings from traditional methods [31]. In our study, parents supervised the testing by coming online first using a family username and password, watching a demonstration and completing a consent form. The twins each had a unique ID number as well as a family number and completed the test in turn. Parents were urged neither to assist the twins with answers nor to allow the twins to see each other’s answers.

### Statistical analyses

All analyses were conducted in OpenMx [32]. This program deals with incomplete data (missing at random) by calculating the log likelihood of the data for each observation using raw maximum likelihood estimation. Standard regression-based corrections for age and sex were applied to raw scores [33] and residual scores were analysed. Means and variances within traits, as well as phenotypic correlations across traits, were equated across twins in a pair and zygosity group in order to generate phenotypic correlation coefficients representative of the entire sample while taking the non-independence of the data into account (i.e. data from related individuals). Separate analyses were conducted for vocabulary and Raven’s scores.

#### The cross-lagged twin model

**Fig 1:**
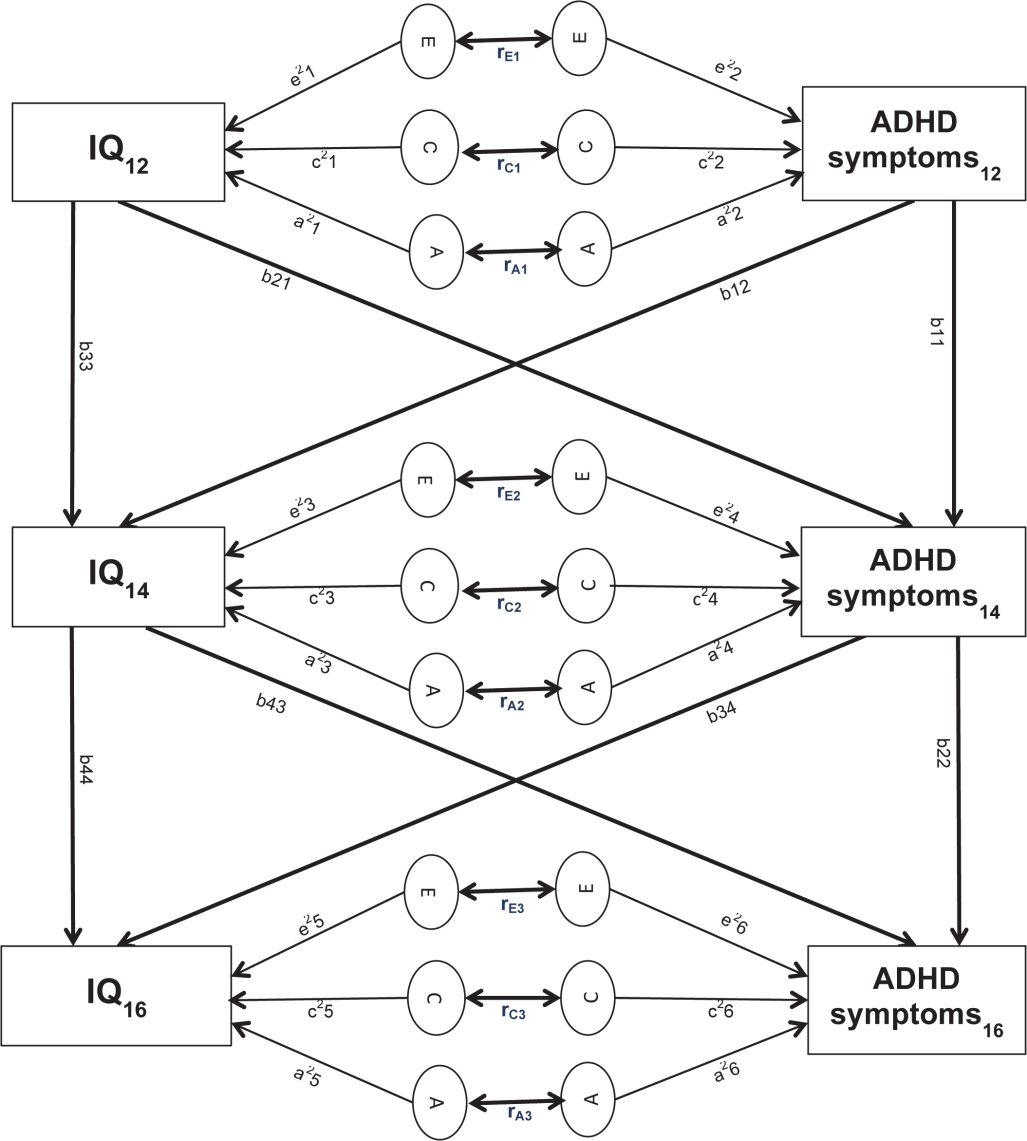
Cross-lagged twin model. Circles represent latent genetic (*A)*, shared environmental (*C*) and non-shared environmental (*E*) factors for ADHD symptoms and IQ at time 1 (age 12), time 2 (age 14) and time 3 (age 16). Paths from the latent A, C, E factors to the observed variables (a1–a6, c1–c6, e1–e6) represent genetic and environmental contributions to ADHD symptoms and IQ scores. The environmental and genetic correlations between ADHD symptoms and IQ for each time point are indicated below the arrows connecting the respective circles (rE, rC, rA). Stability paths (b_11_, b_22_, b_33_, b_44_) connect the same traits across time. Cross-lagged paths (b_12_, b_21_, b_34_, b_43_) connect different traits across time.

*Time-specific effects*: The applied model specifies the time-specific genetic and environmental components of (co)variance in a standard bivariate genetic model using biometrical genetics theory [34]. Estimation of the additive genetic effects (A), shared environment (C) and non-shared environment (E) for each trait is based on the fact that MZ and DZ twins have different degrees of correlation for the genetic component (1 vs 0.5), but the same degree of correlation for shared (1) and non-shared environmental factors (0). This is due to the expectation that MZ twins share 100% and DZ twins share on average 50% of their inherited DNA sequence; yet both MZ and DZ twins share many aspects of their environment by virtue of being born at the same time and place and growing up in the same family. The MZ:DZ correlation ratio indicates the relative importance of the A, C and E components for each trait. In the bivariate twin analysis, MZ and DZ correlations are compared across traits: i.e., one twin’s ADHD symptoms are correlated with the co-twin’s IQ score. If the cross-trait cross-twin correlations are greater for MZ than for DZ twins, this implies that genetic factors contribute to the phenotypic correlation between the two traits. Fig 1 depicts genetic (A), shared environmental (C) and non-shared environmental (E) influences on ADHD symptoms and IQ. Non-shared environmental influences also include measurement error. Fig 1 also presents genetic and environmental correlations (r_A_, r_C_; r_E_), which can range from −1 to 1, and represent the extent to which genetic and environmental influences on ADHD symptoms and the IQ subtests overlap (regardless of their individual heritabilities). Furthermore, phenotypic correlations can be attributed to genetic and environmental influences. The proportion of the phenotypic correlation between ADHD symptoms and the IQ subtests that is due to genetic influences at age 12 years, for example, can be derived by calculating [(a1 x r_A1_ x a2)/ (phenotypic correlation at age 12 years x100)] (Fig 1).

*Cross-lagged effects*: The model specifies the across-time correlations by means of causal cross-lagged paths, which connect different measures across time points (Fig 1: b12, b21, b34, b43). The stability paths connect the same measure across time points (Fig 1: b11, b22, b33, b44). The stability and cross-lagged paths take the form of partial regression coefficients, which take into account the pre-existing association between ADHD symptoms and IQ, as well as controlling for stability or cross-lagged effects.

In order to establish the extent to which the same or different aetiological factors influence ADHD symptoms and IQ over time, genetic and environmental variances were divided into variance specific to t_2_ and t_3_, and variance transmitted from t_1_ to t_2_, and from t_2_ to t_3_. Variance can be transmitted via the stability paths. The genetic variance transmitted via the stability path to ADHD symptoms from t_1_ to t_2_, for example, is calculated as a2^2^ x b11^2^ (Fig 1). Variance can also be transmitted via the cross-lagged paths. The genetic variance transmitted to ADHD symptoms via the cross-lagged path from t_1_ to t_2_, for example, is calculated as a1^2^ x b21^2^. Furthermore, variance can be transmitted via the covariation of ADHD symptoms and IQ. The genetic variance transmitted to ADHD symptoms via the covariation of ADHD symptoms and IQ from t_1_ to t_2_, for example, is calculated as 2x (b21 x a1 x r_A1_ x a2 x b11). To examine the changes in genetic and environmental aetiologies of the association between ADHD symptoms and IQ, the covariance between ADHD symptoms and IQ was divided into covariance specific to t_2_ or t_3_ and covariance shared with the previous time point.

## Results

### Phenotypic correlations

The phenotypic across-trait correlations between ADHD symptoms and vocabulary scores ranged from -0.16 to -0.24 (Table 1). The phenotypic across-trait correlations between ADHD symptoms and Raven’s scores ranged from -0.18 to -0.22 (Table 2). Phenotypic within-trait across-time correlations were high for ADHD symptoms (range: 0.57–0.77; Tables 1 and 2) and moderate for vocabulary (range: 0.20–0.48; Table 1) and Raven’s scores (range: 0.35–0.63; Table 2).

**Table 1:** Phenotypic correlations between ADHD symptom and vocabulary scores.

	ADHD sympt_1_	Vocabulary_1_	ADHD sympt_2_	Vocabulary_2_	ADHD sympt_3_	Vocabulary_3_
ADHD sympt_1_	1					
Vocabulary_1_	-.19 (-.20/-0.16)	1				
ADHD sympt_2_	.77 (.77/.79)	-.19 (-.21/-.16)	1			
Vocabulary_2_	-.23 (-.27/-.21)	.48 (.45/.50)	-.23 (-.27/-.22)	1		
ADHD sympt_3_	.57 (.56/.58)	-.17 (-.19/-.15)	.74 (.73/.75)	-.24 (-.28/-.23)	1	
Vocabulary_3_	-.16 (-.20/-.14)	.20 (.19/.22)	-.18 (-.23/-.16)	.41 (.38/.44)	-.16 (-.21/-.14)	1

95% confidence intervals are provided in brackets.

**Table 2:** Phenotypic correlations between ADHD symptom and Raven’s Standard Progressive Matrices scores.

	ADHD sympt_1_	Raven’s_1_	ADHD sympt_2_	Raven’s_2_	ADHD sympt_3_	Raven’s_3_
ADHD sympt_1_	1					
Raven’s_1_	-.21 (-.24/-.17)	1				
ADHD sympt_2_	. 76 (.75/.77)	-.21 (-.23/-.19)	1			
Raven’s_2_	-.21 (-.24/-.18)	. 54 (.47/.56)	-.22 (-.26/-.20)	1		
ADHD sympt_3_	. 59 (.57/.60)	-.18 (-.19/-.13)	.77 (.76/.78)	-.20 (-.24/-.18)	1	
Raven’s_3_	-.19 (-.20/-.15)	. 35 (.29/.38)	-.22 (-.24/-.17)	.63 (.60/.65)	-.22 (-.24/-.18)	1

95% confidence intervals are provided in brackets.

### Genetic and environmental aetiologies

**Fig 2:**
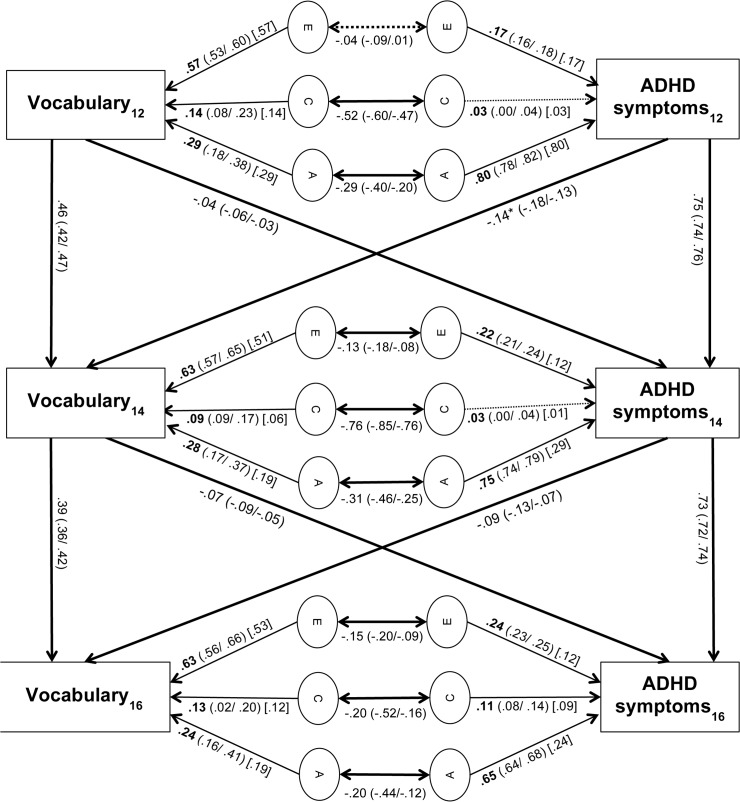
Cross-lagged path model of vocabulary and ADHD symptom scores. 95% confidence intervals are provided in brackets. Values in square brackets [] represent time-specific genetic and environmental contributions to ADHD symptoms and vocabulary scores. Asterisks indicate the significantly greater path at the p = 0.05 level. Non-significant paths are indicated by dashed lines.

**Fig 3:**
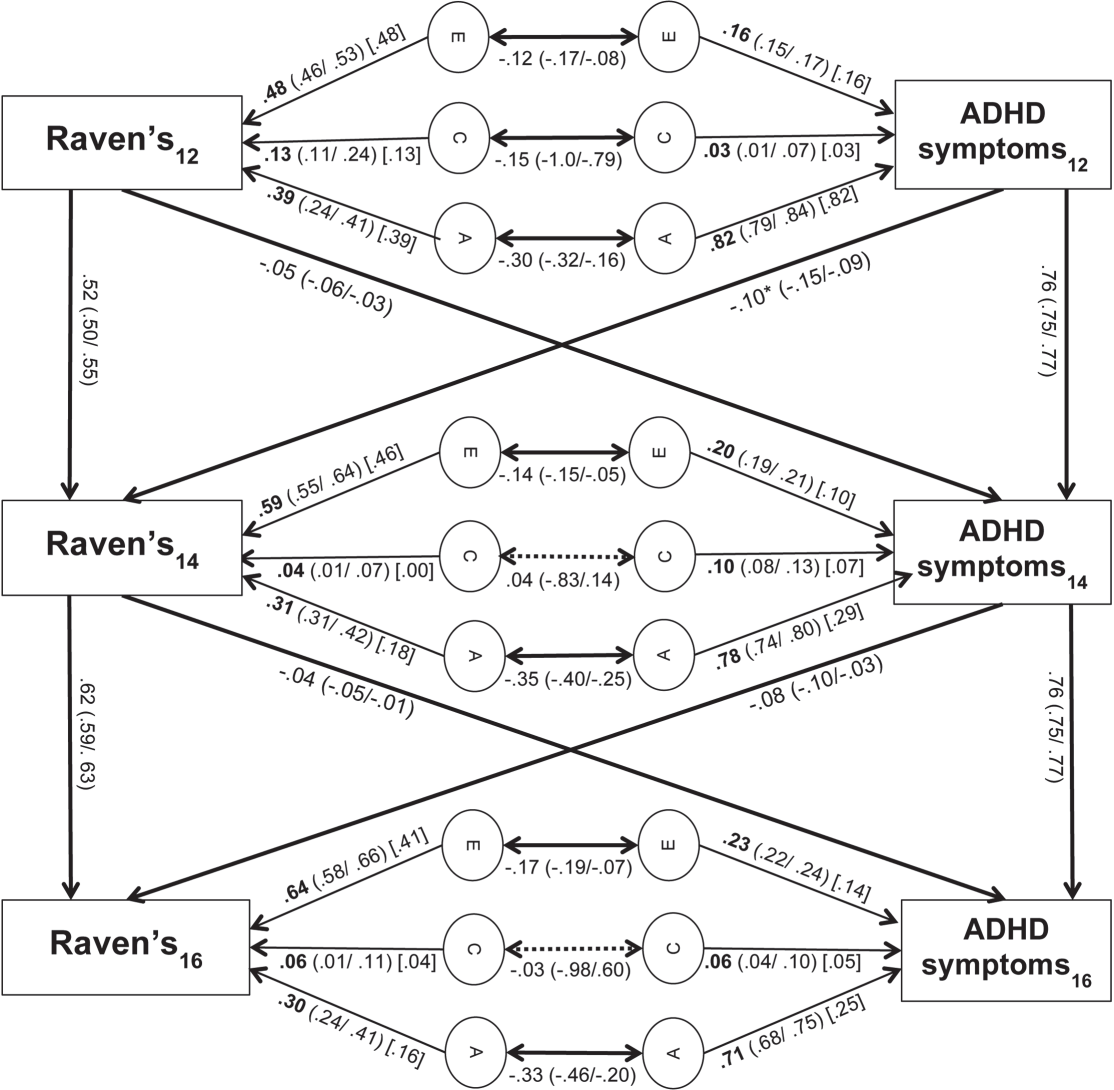
Cross-lagged path model of Raven’s and ADHD symptom scores. 95% confidence intervals are provided in brackets. Values in square brackets [] represent time-specific genetic and environmental contributions to ADHD symptoms and Raven’s Progressive Matrices Scores. Asterisks indicate the significantly greater path at the p = 0.05 level. Non-significant paths are indicated by dashed lines.

Heritabilities of ADHD symptoms were high at each of the three time points (range: 0.65–0.82; Figs 2 and 3). Heritabilities of vocabulary (range: 0.24–0.29; Fig 2) and Raven’s scores (range 0.30–0.39; Fig 3) were moderate at all three time points. The genetic correlation between the total variance of ADHD symptoms and vocabulary scores was significant at each time point (r_A1_ = -0.29, r_A2_ = -0.31, r_A3_ = -0.20; Fig 2). At t_1_, t_2_ and t_3_, 74%, 61% and 50% of the phenotypic correlation between ADHD symptoms and vocabulary scores were attributable to genetic influences, respectively (Table 3). The genetic correlations between the total variance of ADHD symptoms and Raven’s scores were also significant at all three time points (r_A1_ = -0.30, r_A2_ = -0.35, r_A3_ = -0.33, Fig 3). At t_1_, t_2_ and t_3_, 81%, 77% and 68% of the phenotypic correlation between ADHD symptoms and Raven’s scores were attributable to genetic influences, respectively (Table 4).

**Table 3:** Proportions of the phenotypic correlations between ADHD symptoms and vocabulary scores due to genetic (A), shared environmental (C) and non-shared environmental (E) influences.

	Time 1	Time 2		Time 3	
r_ph_	-0.19	-0.23		-0.16	
% of r_ph_ due to		Total^a^	Time-specific^b^	Total^a^	Time-specific^b^
**A**	74% (0.14)	61% (0.14)	30% (0.07)	50% (0.08)	25% (0.04)
**C**	16% (0.03)	17% (0.04)	9% (0.02)	12.5% (0.02)	12.5% (0.02)
**E**	10% (0.02)	22% (0.05)	13% (0.03)	37.5% (0.06)	25% (0.04)

ADHD = attention deficit hyperactivity disorder; r_ph_ = phenotypic correlation

^a^Proportions of the phenotypic correlation due to total genetic or environmental influences (i.e. transmitted plus age-specific effects).

^b^Proportions of the phenotypic correlation due to time-specific genetic or environmental influences specific to early adolescence.

Figures in parentheses refer to the absolute contributions of A, C and E respectively to the phenotypic correlations between ADHD symptoms and vocabulary scores.

**Table 4:** Proportions of the phenotypic correlations between ADHD symptoms and Raven’s Standard Progressive Matrice scores due to genetic (A), shared environmental (C) and non-shared environmental (E) influences.

	Time 1	Time 2		Time 3	
r_ph_	-0.21	-0.22		-0.22	
% of r_ph_ due to		Total^a^	Time-specific^b^	Total^a^	Time-specific^b^
**A**	81% (0.17)	77% (0.17)	36% (0.08)	68% (0.15)	32% (0.07)
**C**	5% (0.01)	0% (0.00)	0% (0.00)	0% (0.00)	0% (0.00)
**E**	14% (0.03)	23% (0.05)	14% (0.03)	32% (0.07)	23% (0.05)

ADHD = attention deficit hyperactivity disorder; r_ph_ = phenotypic correlation

^a^Proportions of the phenotypic correlation due to total genetic or environmental influences (i.e. transmitted plus age-specific effects).

^b^Proportions of the phenotypic correlation due to time-specific genetic or environmental influences specific to early adolescence.

Figures in parentheses refer to the absolute contributions of A, C and E respectively to the phenotypic correlations between ADHD symptoms and Raven’s scores.

For ADHD symptoms, 38–39% of genetic variance (i.e. of the heritability shown in Figs 2 and 3), 33–70% of shared environmental variance, and 50–80% of non-shared environmental variance were due to time-specific aetiological influences at t_2_. At t_3_, 36–79% of genetic variance, 83–92% of shared environmental variance, and 61–84% of non-shared environmental variance were due to time-specific aetiological influences (Tables 5 and 6). For vocabulary scores, 68% of genetic variance, 67% of shared environmental variance and 81% of non-shared environmental variance was attributable to time-specific aetiological influences at t_2_. At t_3_, 79% of genetic variance, 92% of shared environmental variance and 84% of non-shared environmental variance was attributable to time-specific aetiological influences (Table 5). For Raven’s scores, 58% of genetic variance, 0% of shared environmental variance and 78% of non-shared environmental variance was attributable to time-specific aetiological influences at t_2_. At t_3_, 53% of genetic variance, 67% of shared environmental variance and 64% of non-shared environmental variance was attributable to time-specific aetiological influences (Table 6). Thus, many new genetic and environmental influences on ADHD symptoms, vocabulary and Raven’s scores emerged at t_2_ and t_3_, which were not explained by environmental or shared influences at t_1_ or t_2_ respectively.

**Table 5:** Transmission of genetic (A), shared environmental (C) and non-shared environmental (E) influences between ADHD symptoms and vocabulary scores over time.

	Time 2			Time 3		
**ADHD symptoms**	**A**	**C**	**E**	**A**	**C**	**E**
Total ACE variance	0.75	0.03	0.22	0.65	0.11	0.24
Proportion of total ACE variance due to						
*ADHD symptoms (stability effect)*	0.45 (60%)	0.02 (67%)	0.10 (45%)	0.40 (62%)	0.02 (18%)	0.12 (50%)
*Vocabulary scores (cross-lagged effect)*	0.00 (0%)	0.00 (0%)	0.00 (0%)	0.00 (0%)	0.00 (0%)	0.00 (0%)
*Covariation between ADHD symptoms and Vocabulary scores (common effects)*	0.01 (1%)	0.00 (%)	0.00 (%)	0.01 (2%)	0.00 (%)	0.00 (%)
*Time-specific influences*	0.29 (39%)	0.01 (33%)	0.12 (55%)	0.24 (36%)	0.09 (82%)	0.12 (50%)
**Vocabulary scores**	**A**	**C**	**E**	**A**	**C**	**E**
Total ACE variance	0.28	0.09	0.63	0.24	0.13	0.63
Proportion of total ACE variance due to						
*Vocabulary scores (stability effect)*	0.06 (21%)	0.03 (33%)	0.12 (19%)	0.04 (17%)	0.01 (8%)	0.10 (16%)
*ADHD symptoms (cross-lagged effect)*	0.02 (7%)	0.00 (0%)	0.00 (0%)	0.01 (4%)	0.00 (0%)	0.00 (0%)
*Covariation between ADHD symptoms and Vocabulary scores (common effects)*	0.01 (4%)	0.00 (0%)	0.00 (0%)	0.00 (0%)	0.00 (0%)	0.00 (0%)
*Time-specific influences*	0.19 (68%)	0.06 (67%)	0.51 (81%)	0.19 (79%)	0.12 (92%)	0.53 (84%)

*Note*: Percentages in parentheses refer to the proportion of variance at the age indicated transmitted from the previous time point, i.e. transmitted from time 1 to time 2 and from time 2 to time 3. For each trait, percentages within each column add up to 100%, and, thus, may not perfectly correspond to proportions derivable from parameter estimates in this table, due to rounding error.

**Table 6:** Transmission of genetic (A), shared environmental (C) and non-shared environmental (E) influences between ADHD symptoms and Raven’s Standard Progressive Matrices over time.

	Time 2			Time 3		
**ADHD symptoms**	**A**	**C**	**E**	**A**	**C**	**E**
Total ACE variance	0.78	0.10	0.20	0.71	0.06	0.23
Proportion of total ACE variance due to						
*ADHD symptoms (stability effect)*	0.47 (60%)	0.02 (20%)	0.09 (45%)	0.45 (63%)	0.01 (17%)	0.09 (39%)
*Raven’s scores (cross-lagged effect)*	0.01 (1%)	0.01 (10%)	0.01 (5%)	0.00 (0%)	0.00 (0%)	0.00 (0%)
*Covariation between ADHD symptoms and Raven‘s scores (common effects)*	0.01 (1%)	0.00 (0%)	0.00 (0%)	0.01 (1%)	0.00 (0%)	0.00 (0%)
*Time-specific influences*	0.29 (38%)	0.07 (70%)	0.10 (50%)	0.25 (36%)	0.05 (83%)	0.14 (61%)
**Raven‘s scores**	**A**	**C**	**E**	**A**	**C**	**E**
Total ACE variance	0.31	0.04	0.59	0.30	0.06	0.64
Proportion of total ACE variance due to						
*Raven‘s scores (stability effect)*	0.11 (36%)	0.04 (100%)	0.13 (22%)	0.12 (40%)	0.02 (33%)	0.23 (36%)
*ADHD symptoms (cross-lagged effect)*	0.01 (3%)	0.00 (0%)	0.00 (0%)	0.00 (0%)	0.00 (0%)	0.00 (0%)
*Covariation between ADHD symptoms and Raven‘s scores (common effects)*	0.01 (3%)	0.00 (0%)	0.00 (0%)	0.02 (7%)	0.00 (0%)	0.00 (0%)
*Time-specific influences*	0.18 (58%)	0.00 (0%)	0.46 (78%)	0.16 (53%)	0.04 (67%)	0.41 (64%)

*Note*: Percentages in parentheses refer to the proportion of variance at the age indicated transmitted from the previous time point, i.e. transmitted from time 1 to time 2 and from time 2 to time 3. For each trait, percentages within each column add up to 100% and, thus, may not perfectly correspond to proportions derivable from parameter estimates in this table, due to rounding error.

Of the 61% of the phenotypic correlation between ADHD symptoms and vocabulary scores at t_2_ that is due to genetic influences, a proportion of 30% was due to time-specific genetic influences at t_2_, while the remaining 31% were due to lasting genetic influences transmitted from t_1_ (Table 3; 30% + 31% = 61%). At t_3_, 25% of the phenotypic correlation between ADHD symptoms and vocabulary scores were due to time-specific genetic influences, while the remaining 25% were due to lasting genetic influences transmitted from t_2_. A proportion of 9% of the phenotypic correlation between ADHD symptoms and vocabulary scores was due to time-specific shared environmental influences at t_2_, while the remaining 8% were due to lasting shared environmental influences transmitted from t_1_. At t_3_, 12.5% of the phenotypic correlation between ADHD symptoms and vocabulary scores were due to time-specific shared environmental influences, while the remaining 12.5% were due to lasting shared environmental influences transmitted from t_2_. A proportion of 13% of the phenotypic correlation between ADHD symptoms and vocabulary scores was due to time-specific non-shared environmental influences at t_2_, while the remaining 9% were due to lasting non-shared environmental influences transmitted from t_1_. At t_3_, 25% of the phenotypic correlation between ADHD symptoms and vocabulary scores were due to time-specific non-shared environmental influences, while the remaining 12.5% were due to lasting non-shared environmental influences transmitted from t_2_.

Of the phenotypic correlation between ADHD symptoms and Raven’s scores at t_2_, 36% were due to time-specific genetic influences at t_2_, whereas the remaining 41% were due to lasting genetic influences transmitted from t_1_ (Table 4). At t_3_, 32% of the phenotypic correlation between ADHD symptoms and Raven’s scores was due to time-specific genetic influences, whereas the remaining 36% were due to lasting genetic influences transmitted from t_2_. At t_2_ and t_3_, there were no shared environmental influences on the phenotypic correlation between ADHD symptoms and Raven’s scores. A proportion of 14% of the phenotypic correlation between ADHD symptoms and Raven’s scores was due to time-specific non-shared environmental influences at t_2_, while the remaining 9% were due to lasting non-shared environmental influences transmitted from t_1_. At t_3_, 23% of the phenotypic correlation between ADHD symptoms and Raven’s scores were due to time-specific non-shared environmental influences, while the remaining 9% were due to lasting non-shared environmental influences transmitted from t_2_.

Overall, these results indicate that both stable aetiological factors and time-specific genetic and environmental influences emerging for *each trait* at t_2_ and t_3_ (Tables 5 and 6) are involved in the *association* between ADHD symptoms and vocabulary scores and the *association* between ADHD symptoms and Raven’s scores across time.

### Phenotypic cross-lagged effects

All cross-lagged paths were small (Figs 2 and 3). This is a common finding [16,35–37], because cross-lagged paths are partial regression coefficients (i.e. they explain the left-over variance once the within-trait across-time stability has been accounted for). The full predictive relationships between ADHD symptoms and the two indicators of IQ (vocabulary and Raven’s scores) are stronger, as indicated by the phenotypic correlations (Tables 1 and 2 respectively).

The stability paths of ADHD symptoms (b_11_ = 0.75, b_22_ = 0.73) and vocabulary scores (b_33_ = 0.46, b_44_ = 0.39) were significant and moderate to high (Fig 2). All cross-lagged effects from ADHD symptoms to vocabulary scores, and vice versa, were significant, as shown by the 95% confidence intervals. A χ^2^—test indicated that the cross-lagged path from ADHD symptoms at t_1_ to vocabulary scores at t_2_ (b_12_ = -0.14) was significantly larger than the cross-lagged path from vocabulary scores at t_1_ to ADHD symptoms at t_2_ (b_21_ = -0.04; χ^2^ = 67.76, p<0.001, df = 1). The cross-lagged path from ADHD symptoms at t_2_ to vocabulary scores at t_3_ (b_34_ = -0.10) was not significantly different from the cross-lagged path from vocabulary scores at t_2_ to ADHD symptoms at t_3_ (b_43_ = -0.07; χ^2^ = 2.30, p = 0.13, df = 1). Analyses of the relationship between vocabulary and inattention scores, and the relationship between vocabulary and hyperactivity-impulsivity scores, revealed the same pattern as above for both symptom dimensions (not presented here).

In the cross-lagged model of the association between ADHD symptoms and Raven’s scores, the stability paths of ADHD symptoms (b_11_ = 0.76, b_22_ = 0.76) and Raven’s scores (b_33_ = 0.52, b_44_ = 0.61) were similarly significant and moderate to high (Fig 3). All cross-lagged effects from ADHD symptoms to Raven’s scores, and vice versa, were significant, as shown by the 95% confidence intervals. A χ^2^—test indicated that the cross-lagged path from ADHD symptoms at t_1_ to Raven’s scores at t_2_ (b_12_ = -0.10) was significantly larger than the cross-lagged path from Raven’s scores at t_1_ to ADHD symptoms at t_2_ (b_21_ = -0.05; χ^2^ = 15.19, p<0.001, df = 1). The cross-lagged path from ADHD symptoms at t_2_ to Raven’s scores at t_3_ (b_34_ = -0.08) was not significantly different from the cross-lagged path from Raven’s scores at t_2_ to ADHD symptoms at t_3_ (b_43_ = -0.04; χ^2^ = 2.36, p = 0.09, df = 1). Analyses of the relationship between Raven’s and inattention scores, and the relationship between Raven’s and hyperactivity-impulsivity scores, revealed the same pattern as above for both symptom dimensions (not presented here).

## Discussion

In this cross-lagged analysis exploring the developmental patterns of the causal directions and the aetiology of the association between ADHD symptoms and IQ, three key findings emerged that together shed light on the aetiology of the longitudinal relationship between ADHD symptoms and IQ in the general population.

Firstly, time-specific genetic and environmental influences emerged for *each trait* at t_2_ and t_3_. Yet, heritabilities remained high for ADHD and moderate for vocabulary and Raven’s scores across the three time points. This finding suggests that ADHD and IQ are developmentally complex phenotypes characterised by both continuity and change of the aetiological influences across adolescence. The stable and dynamic nature of the genetic risks supports a “developmentally dynamic” hypothesis of ADHD [38]. Considering ADHD in a developmental framework implies that early intervention could help to reduce ADHD symptoms, as well as the impact these symptoms may have on IQ. Future research should investigate the developmental patterns of the association between ADHD symptoms and IQ at earlier as well as later stages of development.

Secondly, we found that the (genetic) association of ADHD symptoms with vocabulary and Raven‘s scores at t_2_ and t_3_ were determined by both time-specific genetic and environmental influences emerging for *each trait* at t_2_ and t_3_ and the associations transmitted from previous time points. Thus, the aetiological factors involved in the *association* of ADHD symptoms with vocabulary and Raven’s scores were moderately stable across time points, despite the large sample and, thus, increased power to detect time-specific aetiological influences. The stable aetiological influences on the association of ADHD symptoms with IQ may indicate that enduring molecular, and hence neurobiological, processes play an important role for the association. Future research should explore these processes, for example through molecular genetic, neuropsychological and neuroimaging studies.

Thirdly, ADHD symptoms and vocabulary scores, and ADHD symptoms and Raven’s scores, significantly predicted each other from t_1_ to t_2_ and from t_2_ to t_3_. However, ADHD symptoms at t_1_ were a significantly stronger predictor of both vocabulary and Raven’s scores at t_2_ than vice versa. No differential effects emerged for the cross-lagged paths from ADHD symptoms at t_2_ to vocabulary or Raven’s scores at t_3_ and vice versa. The lack of differential effects may be explained by the fact that the direct effects of ADHD symptoms at t_2_ on vocabulary or Raven’s scores at t_3_ and vice versa are likely attenuated by the indirect effects from the associations at previous time points (which also explain the relationships). The developmental patterns for the association of ADHD symptoms with these two IQ subscales were highly comparable.

The cross-lagged effects are small, as is common [16,35–37], because cross-lagged paths explain the left-over variance once the within-trait across-time stability has been accounted for. As indicated by the phenotypic correlations, the full predictive relationships between ADHD symptoms and the two indicators of IQ (vocabulary and Raven’s scores) are stronger than the cross-lag estimates. The significant negative cross-lagged associations between ADHD symptoms and the two IQ subscales, therefore, suggest ADHD symptoms could contribute to an increased risk for worse life outcomes, as IQ has previously been shown to predict overall outcome in individuals with ADHD [10,11] and is also a good predictor of life success in the general population, predicting, for example, educational achievement and longevity [12,13]. Contrary to studies investigating VIQ and PIQ in individuals diagnosed with ADHD [3–5], no differential effects emerged for VIQ and PIQ in our population-based twin sample. Future research will have to substantiate these findings in the general population and in individuals diagnosed with ADHD in order to establish potential cognitive strengths and weaknesses associated with ADHD to guide treatment and educational programmes.

One way in which ADHD symptoms may affect IQ scores is through education: the core symptoms of ADHD—inattention, impulsivity and hyperactivity—may lead to difficulties in following teachers’ instructions and lesson content and, thus, interfere with opportunities to benefit from education. Data from the prospective, population-based Avon Longitudinal Study of Parents and Children (n = 11,640) show that parent-rated hyperactivity and inattention symptoms measured at age 3 had negative effects on academic outcomes at age 16 [39]. Furthermore, ADHD symptoms were found to negatively affect academic achievement concurrently and longitudinally (after 5 years) in 192 12-year olds from the general population [40]. These studies suggest that high levels of ADHD symptoms predict poor academic outcomes and lower levels of education, which in turn have been associated with lower scores on Wechsler intelligence subtests [41]. Recent research also suggests that the Raven’s Progressive Matrices test is influenced by education [42,43]. Education may, thus, be a putative mechanism via which ADHD symptoms affects IQ scores in the way our results suggest.

The study has some limitations. We investigated the association between ADHD symptoms and IQ in a population-based sample of twins rather than a clinical sample. The associations of ADHD symptoms with VIQ and PIQ are likely to be greater and may differentiate in individuals diagnosed with ADHD [3,4]. However, examining large unselected population samples decreases the risk of possible selection biases associated with clinical samples. Secondly, the heritability estimates for the IQ subscales are lower than previously reported heritability estimates of general cognitive abilities (g). This may be because g is a latent trait generated from multiple measures and here, we separately analysed vocabulary and Raven’s scores as proxies for VIQ and PIQ. In a genome-wide complex trait analysis of this sample the typical pattern of genetic stability and increasing heritability for g were found, with DNA-estimated heritabilities increasing from 0.26 at age 7 to 0.45 at age 12 years, [44]. Thirdly, there was a change in the specific test used to measure vocabulary from age 14 to 16 years. Yet, the test conducted at age 16 was analogous to the test conducted at ages 12 and 14 years; in both the participant’s task was to select the correct synonym for a word from a list of alternatives. Their similarity is reflected in the comparable magnitudes of the stability paths from ages 12–14 years and ages 14–16 years. Lastly, while a cross-lag model may allow us to examine the direction of effects, further research is required to draw firm conclusions about the direction of causality.

These limitations notwithstanding, the prospective developmental design of our cross-lagged analysis allowed us to explore the direction of effects for the negative association between ADHD symptoms and IQ scores, as well as the dynamic and stable nature of the aetiological influences on these variables and their association. ADHD symptoms and IQ scores significantly predicted each other over time. Yet, ADHD symptoms at age 12 years were a significantly stronger predictor of both vocabulary and Raven’s scores at age 14 years than vice versa. Time-specific aetiological influences emerged for each trait. However, the aetiological factors involved in the association between ADHD symptoms and IQ were highly stable over time. Future research will need to build on these results to investigate how early intervention targeting ADHD symptoms may aid in reducing the risk for lower IQ.

## Supporting Information

S1 Dataset Data from the Twins Early Development Study: ADHD symptom ratings, Vocabulary and Raven’s Progressive Matrices score at age 12, 14 and 16. (CSV)
